# Self-perceived oral health and orofacial appearance in older adults – an 18-year follow-up study in Karlskrona, Sweden

**DOI:** 10.2340/aos.v83.40574

**Published:** 2024-05-03

**Authors:** Sara Henricsson, Viveca Wallin Bengtsson, Stefan Renvert, Johan Sanmartin Berglund, Nina Lundegren, Pia Andersson

**Affiliations:** a Faculty of Health Sciences, Kristianstad University, Kristianstad, Sweden; b Faculty of Odontology, Malmö University, Malmö, Sweden; c Department of Health, Blekinge Institute of Technology, Karlskrona, Sweden

**Keywords:** Older adults, oral health, orofacial appearance, self-perception

## Abstract

**Objectives:**

To analyze whether self-perceived oral health and orofacial appearance change with increasing age.

**Methods:**

This longitudinal study is based on data from a questionnaire used in the Swedish National Study of Aging and Care. The sample comprises 160 participants 60 years of age at baseline 2001–2003. The same participants were re-examined at 66-, 72-, and 78 years of age. To analyze whether perceptions of oral health and orofacial appearance changed with increasing age, Cochran’s Q test was conducted. Statistical significance was considered at *p* ≤ 0.05, and the calculated value Q must be equal to or greater than the critical chi-square value (*Q* ≥ 7.82). Significance values have been adjusted for the Bonferroni correction for multiple tests.

**Results:**

Self-perceived mouth dryness, both day (*Q* = 7.94) and night (*Q* = 23.41), increased over the 18-year follow-up. When divided by gender, significant differences were only seen for mouth dryness at nighttime. A decrease in sensitive teeth was perceived with increasing age, and an increase in self-perceived satisfaction with dental appearance, and a decrease in self-perceived problems with dental gaps between the ages of 60 and 78. These changes were, however, not statistically significant. Men experienced a higher proportion of discomfort with discolored teeth at age 78 than at 60 (*Q* = 9.09).

**Conclusions:**

Self-perceived oral health and orofacial appearance were relatively stable, with few changes over an 18-year follow-up.

## Introduction

Oral health is important at any age and, together with a pleasing facial appearance, contributes to the quality of life [1, 2]. For older adults, good oral health and the retention of natural teeth are beneficial for oral functions [1]. Deteriorating oral health may lead to restricted participation in social activities [1, 2].

Older adults are a growing group within the population [3], and this development is expected to continue both in Sweden [4] and in other countries [3]. Increased life expectancy can be seen as a success, but it also brings challenges, and with an ageing population, the risk of diseases may increase [5]. It is not clear where ‘normal’ ageing ends and diseases begin. Older adults are physiologically a heterogeneous group and differ more from one another than younger adults [6, 7].

Besides the steadily growing older adult population, the retention of natural teeth has increased in Sweden [8] and in many other parts of the world [9]. While an increased number of natural teeth is considered an important indicator of a population’s oral health [10], it also poses certain challenges [11]. Natural teeth combined with complex prosthetic constructions [12] place high demands on older adults’ ability to perform oral hygiene [13]. Impaired fine motor skills, for example, can limit the ability to perform and maintain adequate oral hygiene [13, 14]. Together with the presence of gingival retractions, reduced salivary flow, and insufficient oral hygiene, the risk of dental caries and periodontal disease may increase [15]. Reduced salivary flow is not related to age alone. Certain medicines and general diseases may, however, increase the risk of xerostomia in older adults [16].

Normal age-related changes in the oral cavity occur gradually with time. Abrasion and attrition, for instance, result from wear and tear from mastication [7, 17]. Alterations in the enamels’ molecular composition lead to increased brittleness of the teeth, resulting in cracks along the enamel surface [17, 18]. Dentin also transforms with age. Due to the ingrowth of secondary dentin, the pulp chamber decreases in size and sclerosis of dentinal tubules reduces sensitivity to hot and cold [17, 19]. Furthermore, the oral mucosa becomes less resilient, partially due to the loss of elastic fibers and disarray of collagen in the connective tissue, leading to impaired wound healing [19]. These age-related changes in the oral cavity also contribute to visible orofacial changes in appearance that occur with age. Intrinsic discoloration occurs due to alterations in the composition or thickness of the hard dental tissue. The natural ingrowth of secondary dentin affects the light-transmitting properties of teeth, causing them to darken gradually with age [18–20]. Extrinsic staining derives from dietary sources or from something habitually placed in the mouth. Further, abrasion, attrition and staining of fractures and cracks along the enamel surface also contribute to changes of the orofacial appearance [18, 19].

Good oral health is important and affects general health and quality of life [1, 9]. Good self-perceived oral health also seems to correlate with good general health [21, 22]. Self-perceived oral health refers to the individual’s experience of how oral health affects oral function and, as a consequence, social well-being [23]. The association between oral health and mental state is also accounted for in the psychosocial function, one of the core elements of the theoretical framework for the definition of oral health [24]. A previous study on self-perceived oral health and orofacial appearance showed that older adults experience their oral health and orofacial appearance as satisfactory [25]. However, research on how older adults perceive their oral health and orofacial appearance over time is scarce. In contrast, health-related changes over time have been examined, showing an increase in higher levels of positive self-rated health [26]. Research on body appearance suggests that the body image of older adults can have important implications for their well-being [27]. Growing older does not mean that bodily appearance becomes less important [28], which also applies to orofacial appearance. Following the same individuals over time can provide insight into whether perceptions change with increasing age. The present study aims to analyze whether self-perceived oral health and orofacial appearance change with increasing age.

## Methods

### Study design

This longitudinal study is based on survey data from a questionnaire used in the Swedish National Study of Aging and Care (SNAC). The study was approved by the Research Ethics Committee at Lund University, Sweden (No: LU 604/00) and conducted in accordance with the World Medical Association Declaration of Helsinki [29]. Signed informed consent was obtained, and the collected data were anonymously processed.

### Context

SNAC is a population-based longitudinal cohort study, initiated in 2001 to capture and study aging from the transition between work to retirement and higher age [30]. Karlskrona, a municipality in southeastern Sweden, is one of four participating centers [30], and the only center where oral health is studied. The participating municipalities in SNAC represent different geographical regions, and Karlskrona represents a medium-sized city [30]. Subjects were selected from the Swedish civil registration database in Karlskrona in the age group of 60-96 years. Individuals aged 60, 66, 72, and 78 were randomly selected, whereas all individuals aged 81, 84, 87, 90, 93, and 96 years (and older) were invited to participate. The data collection is ongoing and invites new 60- and 81-year-olds to participate every 6 years. At baseline 2001–2003, approximately 10% of the inhabitants represent the sample in the studied community. The subjects were invited to participate in a medical, psychological, and oral health examination and were asked to complete a questionnaire.

### Sample and data collection

This study comprises the participants who were 60 in 2001–2003 until the age of 78. In total, 263 60-year-old individuals were randomly selected at baseline 2001–2003. Seventy-two (27.4%) declined participation. Eight of the included 191 participants enrolled at baseline did not answer the questionnaire and were excluded, leaving 183 participants. The participants were re-examined in 2007–2009, 2014–2015, and 2019–2021. In addition to baseline registrations, the participants had to attend at least one follow-up and answer the oral health questionnaire at the follow-up. If participants were unable to visit the research clinic, there was an opportunity for a home visit and/or to complete the questionnaire via a telephone interview. Twenty-three participants attended only at baseline, leaving 160 participants, 90 of whom participated at baseline and all three follow-ups.

### Questionnaire

The oral health questionnaire used in SNAC-Blekinge is based on questions deriving from validated questionnaires [31, 32] and has previously been described [25]. The response alternatives to questions with more than two response alternatives were dichotomized (Table 1).

**Table 1:** Presenting the questions from the SNAC questionnaire that were included in this study and how they were dichotomized according to response options and coded.

Question	Item	Answer	Dichotomization
Country of birth?	Place of birth	1 = Sweden 2 = Other Norden countries, 3 = EU except the Norden countries, 4 = Europe except EU/Norden countries, 5 = Outside Europe	1 ‘Sweden = 1’ 2–5 ‘Other country = 2’
Who lives with the participant?	Living arrangement	1 = Lives alone 2 = Together with spouse, 3 = Daughter, 4 = Son, 5 = Grandchild, 6 = Sibling, 7 = Sister/brother-in-law, 8 = Other	1 ‘Lives alone = 0’ 2–8 ‘Living with someone = 1’
What is your level of education?	Level of Education	1 = Incomplete/unfinished elementary school 2 = Up to elementary school 3 = Elementary school 4 = Upper secondary school 5 = Vocational education 6 = Education at least one year in college or university without a degree 7 = University/College with degree 8 = Postgraduate education	1–3 ‘≤ 9 year of schooling = 0’ 4–8 ‘˃ 9 year of schooling = 1’
Are you satisfied with the appearance of your teeth?	Satisfaction with appearance?	1 = Yes, very satisfied 2 = Yes, pretty much satisfied 3 = No, not particularly satisfied 4 = No, dissatisfied	1–2 ‘Satisfied = 1’ 3–4 ‘Dissatisfied = 0’
Do you avoid contacting other people due to teeth problems?	Avoid contact due to problem with teeth	1 = Yes, to a great extent 2 = Yes, to some extent 3 = Don’t know 4 = No	1–3 ‘Yes = 0’ 4 ‘No = 1’
Do you feel dry in your mouth at day/night?	Mouth dryness (daytime) Mouth dryness (nighttime)	1 = Yes, often 2 = Yes sometimes 3 = No, never	1–2 ‘Yes = 0’ 3 ‘No = 1’
You can have many different problems from your mouth and teeth. Do you experience any of the following concerns? discomfort or concern with;	Discolored teeth Bleeding from the gums Sensitive teeth Tooth mobility Oral halitosis Burning mouth syndrome Tongue coatings Soreness/pain when chewing Dental gaps Difficulty to open mouth Mouth ulcers	1 **=** No discomfort 2 = Some discomfort 3 = Fairly large discomfort 4 = Large discomfort	1 ‘No discomfort = 1’ 2–4 ‘Discomfort = 0’

SNAC: Swedish National Study of Aging and Care.

### Statistical analysis

The IBM SPSS version 28.0.1.0 was used for descriptive and analytical statistics. Descriptive statistics with frequency distribution were summarized concerning dichotomous data based on time intervals for 60 years at baseline and 66, 72, and 78 years.

To analyze whether perceptions of oral health and orofacial appearance change with increasing age, Cochran’s Q test were conducted to determine whether there were differences in a dichotomous dependent variable between three or more related groups [33]. Pair-wise Cochran’s Q tests were performed to identify areas with differences. Statistical significance was considered at *p* ≤ 0.05, and the calculated value Q must be equal to or greater than the critical chi-square value (*x*^2^(3) = *Q*) [33, 34] of 7.82 (*Q* ≥ 7.82). Significance values were adjusted for multiple tests using the Bonferroni correction [35].

## Results

### Background characteristics

Gender distribution remained relatively unchanged to the 18-year follow-up (females ranging from 53.3 to 47.5%). At baseline, 51.2% of participants had an educational attainment of >9 years. Living arrangements changed with increasing age, where the number of those living alone increased (Table 2). Although more women than men lived alone, the number of both men (*x*^2^(3) = 95.16) and women (*x*^2^(3) = 24.62) who lived alone increased significantly with age (Table 3).

**Table 2:** Cross-sectional data on background characteristics of the individuals at 60, 66, 72, and 78 years in total and gender differences (n, %).

Background characteristics	60yr *n* = 160 %	F♀ *n* = 83 %	M♂ *n* = 77 %	66yr *n* = 156 %	F♀ *n* = 79 %	M♂ *n* = 77 %	72yr *n* = 135 %	F♀ *n* = 72 %	M♂ *n* = 63 %	78yr *n* = 99 %	F♀ *n* = 47 %	M♂ *n* = 52 %
Level of Education												
≤ 9 years of schooling	48.8	53.0	44.2									
˃ 9 years of schooling	51.2	47.0	55.8									
Living arrangement												
Lives alone	15.6	19.3	11.7	20.5	25.3	15.6	21.9^a^	32.8^b^	9.8^c^	31.0^d^	45.8^e^	17.3
Living with someone	84.4	80.7	88.3	79.5	74.7	84.4	78.1	67.2	90.2	69.0	54.2	82.7
Cash margin, Can you,												
within a week, get												
14,000 SEK^†^												
No	11.2	14.5	7.8	7.1	11.4	2.6	8.7^f^	13.6^g^	3.3^c^	10.0^d^	12.5^e^	7.7
Yes	88.8	85.5	92.2	92.9	88.6	97.4	91.3	86.4	96.7	90.0	87.5	92.3

† From 2007, the amount was SEK 15,000 and from 2019, the amount was SEK 17,000 adjusted for Consumer Price Index.

a *n* = 128,

b *n* = 67,

c *n* = 61,

d *n* = 100,

e *n* = 48,

f *n* = 127,

g *n* = 66.

**Table 3:** Changes over time in background characteristics from 60 years and at 66, 72, and 78 years in total and gender differences (n).

Background characteristics	Q^c^		Pairwise comparison Adj. Sig.^ab^					
			60–66yr *N*	60–72yr *N*	60–78yr *N*	66–72yr *N*	66–78yr *N*	72–78yr *N*
Living arrangement	**108.78** ^a^		**156** ^a,b^	**128** ^a,b^	**100** ^a,b^	124	97	95
Lives alone		♀	**79** ^a,b^	**67** ^a,b^	**48** ^a,b^	63	45	47
Living with someone		♂	**77** ^a,b^	**61** ^a,b^	**52** ^a,b^	61	52	48
Cash margin, Can you,	6.60		156	127	100	123	97	94
within a week, get		♀	79	66	48	66	45	46
14,000 SEK^†^		♂	77	61	52	61	52	48
No								
Yes								

† From 2007, the amount was SEK 15,000 and from 2019, the amount was SEK 17,000 adjusted for Consumer Price Index.

a *p* < 0.05; Cochran´s Q test.

b Significance values have been adjusted by the Bonferroni correction for multiple tests.

c Q ≥ 7.82 for the null hypothesis to be rejected.

### Self-perceived oral health and orofacial appearance

**Figure 1:**
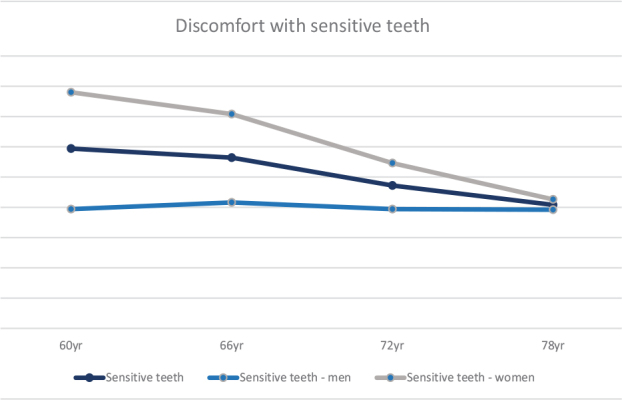
Associations of discomfort with sensitive teeth together and divided by gender over time from baseline 2001–2003 at 60 years and at 66, 72, and 78 years (%).

Self-perceived mouth dryness, both day and night, increased during the 18-year follow-up (Tables 4 and 5). When studying gender separately, significant differences were only seen for mouth dryness at nighttime. For both men (*x*^2^(3) = 17.69) and women (*x*^2^(3) = 8.46), the significance remained after follow-up pair-wise comparisons adjusted for Bonferroni (Table 5). A decrease in sensitive teeth was perceived with increasing age but not significant (Figure 1). In women, the reduction was statistically significant (*x*^2^(3) = 11.54), but when follow-up pair-wise comparisons were adjusted for Bonferroni, the difference in women was no longer significant (Table 5).

**Table 4:** Cross-sectional data on self-reported oral health at 60-, 66-, 72- and 78-years in total and gender differences (n, %).

Self-reported oral health items	60yr			66yr			72yr			78yr		
	*n* = 158%	F♀ *n* = 82 %	M♂ *n* = 76 %	*n* = 156 %	F♀ *n* = 79 %	M♂ *n* = 77 %	*n* = 127 %	F♀ *n* = 66 %	M♂ *n* = 61 %	*n* = 98%	F♀ *n* = 47 %	M♂ *n* = 51 %
Bleeding gums												
No discomfort	76.6	74.4	78.9	76.9	74.7	79.2	72.4	72.7	72.1	79.6	80.9	78.4
Tooth mobility												
No discomfort	91.8^a^	92.8^b^	90.8	94.2	96.2	92.2	92.1	93.9	90.2	90.6^c^	97.8^d^	84.0^e^
Sensitive teeth												
No discomfort	70.3	61.0	80.3	71.8	64.6	79.2	76.4	72.7	80.3	79.6	78.7	80.4
Difficulty to open												
the mouth												
No discomfort	95.6	92.7	98.7	95.5	93.7	97.4	96.9	97.0	96.7	96.9	97.9	96.1
Soreness/pain												
when chewing												
No discomfort	92.4	90.2	94.7	92.9	93.7	92.2	93.7	95.5	91.8	95.9	95.7	96.1
Burning mouth												
syndrome												
No discomfort	92.4	89.0	96.1	94.2	96.2	92.2	93.7	90.9	96.7	93.9	91.5	96.1
Mouth dryness												
daytime												
No discomfort	67.7	57.3	78.9	64.7	63.3	66.2	61.9^f^	56.9^g^	67.2	53.9^h^	45.5^i^	62.2^j^
Mouth dryness												
nighttime												
No discomfort	52.6^k^	41.6^l^	63.6^l^	38.5	35.4	41.6	31.0^f^	27.3	35.0^m^	26.1^n^	25.0^i^	27.1^i^
Cold sores												
No discomfort	83.5	78.0	89.5^l^	82.1	77.2	87.0	88.2	84.8	91.8	96.9	97.9	96.1

a n=159,

b n=83,

c n=96,

d n=46,

e n=50,

f n=126,

g n=65,

h n=89,

i n=44,

j n=45,

k n=154,

l n=77,

m n=60,

n n=92.

**Table 5:** Changes over time in self-reported oral health at 60 years and at 66, 72, and 78 years in total and gender differences (n).

Self-reported oral health items	Q^c^		Pairwise comparison Adj. Sig.^ab^					
			60–66yr *N*	60–72yr *N*	60–78yr *N*	66–72yr *N*	66–78yr *N*	72–78yr *N*
Bleeding gums	3.53		154	125	97	123	95	92
No discomfort	3.40	♀	78	65	46	62	44	45
	1.50	♂	76	60	51	61	51	47
Tooth mobility	1.91		153	123	96	122	94	90
No discomfort	3.00	♀	77	64	46	62	44	45
	3.00	♂	76	59	50	60	50	45
Sensitive teeth	6.42		154	125	97	123	95	92
No discomfort	**11.54** ^a^	♀	78	65	46	62	44	45
	1.94	♂	76	60	51	61	51	47
Difficulty to open	0.92		154	125	97	123	95	92
the mouth	0.86	♀	78	65	46	62	44	45
No discomfort	1.00	♂	76	60	51	61	51	47
Soreness/pain	3.86		154	125	97	123	95	92
when chewing	6.14	♀	78	65	46	62	44	45
No discomfort	3.00	♂	76	60	51	61	51	47
Burning mouth	0.94		154	125	97	123	94	92
syndrome	6.00	♀	78	65	46	62	43	45
No discomfort	1.74	♂	76	60	51	61	51	47
Mouth dryness	**7.94** ^a^		154	124	87	122	86	83^ab^
daytime	6.42	♀	78	64	43	61	41	41
No discomfort	5.78	♂	76	60	44	61	45	42
Mouth dryness	**23.41** ^a^		151^ab^	122^ab^	90^ab^	122	90	86
nighttime	**8.46** ^a^	♀	74	62^ab^	42	62	42	42
No discomfort	**17.69** ^a^	♂	77^ab^	60^ab^	48^ab^	60	48	44
Cold sores	**15.54** ^a^		154	125	97^ab^	123	95^ab^	92
No discomfort	**12.50** ^a^	♀	78	65	46^ab^	62	44^ab^	45
	6.80	♂	76	60	51	61	51	47

a *p* < 0.05; Cochran’s Q test.

b Significance values have been adjusted by the Bonferroni correction for multiple tests.

c *Q* ≥ 7.82 for the null hypothesis to be rejected.

Self-perceived satisfaction with dental appearance increased with age (Tables 6 and 7). Between the ages of 60 and 78, there were significant differences in increased self-perceived satisfaction with dental appearance (*p* = 0.048) and a decrease in self-perceived problems with dental gaps (*p* = 0.043) (not shown in Table). However, these differences over time were no longer significant after adjustments for Bonferroni. A higher proportion of men experienced discomfort with discolored teeth at age 78 than at age 60 (*x*^2^(3) = 9.09). Discomfort with self-perceived oral halitosis decreased in the total population between the ages of 60 to 78 years. Self-perceived oral halitosis among women decreased between the ages of 60 and 78 (*x*^2^(3) = 10.26) and increased among men between the ages of 60 and 72 although not significantly (Table 7).

**Table 6:** Cross-sectional data on self-reported concerns with orofacial appearance at 60, 66, 72, and 78 years in total and gender differences (n, %).

Self-reported orofacial appearance items	60yr *n* = 158 %	F♀ *n* = 81 %	M♂ *n* = 76 %	66yr *n* = 156 %	F♀ *n* = 79 %	M♂ *n* = 77 %	72yr *n* = 127 %	F♀ *n* = 66 %	M♂ *n* = 61 %	78yr *n* = 97 %	F♀ *n* = 47 %	M♂ *n* = 50 %
Satisfaction with												
dental appearance												
Satisfied	78.1^a^	71.1^b^	85.7^c^	82.6^d^	84.8	80.3^e^	89.0	92.4	85.2	88.7	89.4	88.0
Discolored teeth												
No discomfort	72.0^f^	64.2	80.3	72.4	69.6	75.3	74.8	75.8	73.8	69.4^g^	67.4^h^	71.2^i^
Dental gaps												
No discomfort	90.4^f^	91.4	89.5	87.8	88.6	87.0	89.7^j^	90.9	88.3^k^	82.5	85.1	80.0
Oral halitosis												
No discomfort	75.9	73.2^l^	78.9	78.8	83.5	74.0	79.5	86.4	72.1	83.7^g^	91.5	76.5^m^
Tongue coating												
No discomfort	91.1	89.0^l^	93.4	87.8	87.3	88.3	86.6	83.3	90.2	87.6	87.0^h^	88.2^m^
Avoid contact due to												
problems with teeth												
No discomfort	99.4	100.0	98.7^c^	100.0	100.0	100.0	99.2	100.0	98.4	99.0	97.9	100.0

a *n* = 160,

b *n* = 83,

c *n* = 77,

d *n* = 155,

e *n* = 76,

f *n* = 157,

g *n* = 98,

h *n* = 46,

i *n* = 52,

j *n* = 126,

k *n* = 60,

l *n* = 82,

m *n* = 51.

**Table 7:** Changes over time in self-reported concerns with orofacial appearance from 60 years and at 66, 72, and 78 years in total and gender differences (n).

Self-reported orofacial appearance items	Q^c^		Pairwise comparison Adj. Sig.^ab^					
			60–66yr *N*	60–72yr *N*	60–78yr *N*	66–72yr *N*	66–78yr *N*	72–78yr *N*
Satisfaction with	4.60		155	127	97	122	93	91
dental appearance	7.24	♀	79	66	47	62	44	45
Satisfied	0.71	♂	76	61	50	60	49	46
Discolored teeth	5.03		153	125	97	123	95	93
No discomfort	0.42	♀	77	65	44	62	43	45
	**9.09** ^a^	♂	76	60	52^ab^	61	52	48
Dental gaps	4.83		153	123	96	122	94	90
No discomfort	0.75	♀	77	64	46	62	44	45
	5.70	♂	76	59	50	60	50	45
Oral halitosis	3.35		154	125	97	123	95	92
No discomfort	**10.26** ^a^	♀	78	65	46^ab^	62	44	45
	2.90	♂	76	60	51	61	51	47
Tongue coating	1.27		154	125	96	123	94	91
No discomfort	0.53	♀	78	65	45	62	43	44
	0.86	♂	76	60	51	61	51	47
Avoid contact due to	3.00		155	125	95	123	94	91
problems with teeth	^–^	♀	–	–	45	–	44	45
No discomfort	3.00	♂	77	61	50	61	–	46

a *p* < 0.05; Cochran’s Q test.

b Significance values have been adjusted by the Bonferroni correction for multiple tests.

c *Q* ≥ 7.82 for the null hypothesis to be rejected.

## Discussion

The principal findings in this study were that changes in self-perceived oral health and orofacial appearance do not change substantially with increasing age.

The findings make it easy to conclude that only participants who were healthy remained in the study until the age of 78. Out of the 98 participants in the study, only 58 visited the research clinic 2019–2021. The remaining forty 78-year-olds answered the questionnaire during a telephone interview. It is worth noting that the 18-year follow-up (2019–2021) occurred during the COVID-19 pandemic, which may explain why participants did not come to the research clinic. The questions in the questionnaire were derived from validated instruments. A methodological aspect to consider is that the questionnaire form used has not been validated, posing a risk in reducing the validity of the findings. In the present study, orofacial appearance is referred to in the sense that the perceptions of self-perceived oral health problems and/or esthetic appearance may influence and/or have an impact on the perception of orofacial appearance [36].

The study is a population-based longitudinal cohort study and, as mentioned earlier, constitutes 10% of Karlskrona’s population in the different age cohorts randomly selected for recruitment at the time of the surveys. With a limited sample size comes the risk of an ‘underpowered’ study and that of an actual difference will not be observed. However, although the number of 78-year-old individuals re-examined in 2019–2021 had decreased, it still represents 10% of Karlskrona’s 78-year-olds. The low number of participants in the study may have contributed to the lack of significant results. Even so, the gender distribution also corresponds to that of 60-, 66-, 72-, and 78-year-olds in Karlskrona at the time of the survey [37]. As mentioned earlier, Karlskrona represents a medium-sized city and thus can only be generalized as such [30]. In the present study, it is not unreasonable to assume that this generalization also includes gender differences.

Fine motor skills deteriorate with age [14], and age-related changes in the oral cavity [7, 17–19] and orofacial appearance [18–20] may lead to implications in the forms of oral diseases or dissatisfaction with orofacial appearance. Nevertheless, the findings show that participants self-perception were satisfactory for most items surveyed and did not change significantly over time. One plausible explanation is that older adults have been shown to experience more emotional well-being [38], and that aging is associated with increased self-perceived well-being and reduced depressive symptoms compared to younger adults [39]. Another explanation could be that people with higher self-esteem tend to assess their health as more positive [40]. In the present study, neither emotional well-being nor high self-esteem was investigated, but this could explain why participants did not experience a more significant difference in oral health or orofacial appearance over time. Older adults might be better at dealing with negative emotions because of increased life experience [38].

Self-perceived mouth dryness during the daytime increased from baseline to the 18-year follow-up. In agreement with the present study, Johansson et al. [41] and Åstrøm et al. [42] found self-reported mouth dryness at nighttime to increase with age. Johansson et al. [41] also reported concordant findings regarding self-reported mouth dryness during the daytime. In contrast, Åstrøm et al. [42], who performed a cross-national study concerning Sweden and Denmark, presented concordant findings for the Swedish cohort.

The findings in the present study show a higher prevalence of self-perceived mouth dryness in women. A similar magnitude in the prevalence of self-perceived mouth dryness in women at 60 was found in men at 72 years for mouth dryness at nighttime and at 78 years daytime. These findings agree with those of Johansson et al. [41], indicating some gender differences in self-perceived mouth dryness. According to a longitudinal analysis [43] of parotoid and submandibular salivary flow rates, decreased salivary flow rates are not to be considered a normal aging process. As the data analyzed in our study are self-reported, no conclusions can be made about whether the salivary flow rate is reduced. The findings only show the subjective perception of mouth dryness. The increase in self-perceived mouth dryness from age 60 to 78 could be related to side effects from medication. It does, however, not explain the gender difference over time, which also seems to be the case in other studies [41, 42, 44].

The difference in self-perceived oral health concerns between men and women appears to be the same or even decrease with increasing age, except for oral halitosis and tooth mobility. Discomfort with sensitive teeth is one example where the differences in self-reported oral health between men and women decrease with age. Nearly 40% of the women reported discomfort with sensitive teeth at age 60, and by age 78, the figure was close to 20%, while men remained stable at around 20% with minor variations from age 60 to 78. A previous study [25] showed that twice as many 60-year-old women from three different cohorts experienced discomfort from sensitive teeth compared to men. The pulp chamber decreases in size with age [17, 19], which may explain a decrease in sensation and thus pain [17]. Pulpal response time increases and the older the patients are, the lower the pain intensity [45]. This implies that age-related changes in the dentin may, accordingly, explain the lack of discomfort from sensitive teeth although it does not account for why the discomfort from sensitive teeth is not reduced in men over time. One explanation previously discussed [46] is that women are more attentive to their health in general. Therefore, it is possible that men in their 60s are not as prone to express discomfort as women.

The studied individuals perceived good oral health. Ageing is universal although not uniform, but up until 80 years, most people do not have functional impairment or disability [47]. However, older individuals can relatively quickly go from being healthy and active, to frail [48] and the age around 80 seems to be a transitional period when health changes take place [47]. It is therefore important for older individuals not to lose contact with their dental caregiver, which is common with increasing age [49]. Conclusively, changes in self-perceived oral health and orofacial appearance were relatively stable, with few changes over an 18-year follow-up in the studied older population.

## Data Availability

The data supporting the findings of this study are available at reasonable request. The data is not publicly available due to privacy or ethical restrictions.
